# Study and characterization of porous copper oxide produced by electrochemical anodization for radiometric heat absorber

**DOI:** 10.1186/1556-276X-9-577

**Published:** 2014-10-15

**Authors:** Sonia Ben Salem, Zahra Ben Achour, Kamel Thamri, Oualid Touayar

**Affiliations:** 1 Research Laboratory Materials, Measurement and Applications, Institut National des Sciences Appliquées et de Technologie, INSAT, BP676, 1080 Tunis Cedex, Tunisia

**Keywords:** Absorbing cavity, Absolute laser energy, Radiometric measurement, Metrology, Electrochemical anodization, Thermal diffusivity

## Abstract

The aim of this work is to optimize the different parameters for realization of an absorbing cavity to measure the incident absolute laser energy. Electrochemical oxidation is the background process that allowed the copper blackening. A study of the blackened surface quality was undertaken using atomic force microscopy (AFM) analysis and ultraviolet-visible-infrared spectrophotometry using a Shimadzu spectrophotometer. A two-dimensional and three-dimensional visualization by AFM of the formed oxide coating showed that the copper surfaces became porous after electrochemical etching with different roughness. This aspect is becoming more and more important with decreasing current density anodization. In a 2 mol L^-1^ of NaOH solution, at a temperature of 90°C, and using a 16 mA cm^2^ constant density current, the copper oxide formed has a reflectivity of around 3% in the spectral range between 300 and 1,800 nm. Using the ‘mirage effect’ technique, the obtained Cu_2_O diffusivity and thermal conductivity are respectively equal to (11.5 ± 0.5) 10 to 7 m^2^ s^-1^ and (370 ± 20) Wm^-1^ K^-1^. This allows us to consider that our Cu_2_O coating is a good thermal conductor. The results of the optical and thermal studies dictate the choice of the cavity design. The absorbing cavity is a hollow cylinder machined to its base at an angle of 30°. If the included angle of the plane is 30° and the interior surface gives specular reflection, an incoming ray parallel to the axis will undergo five reflections before exit. So the absorption of the surface becomes closely near 0.999999.

## Review

### Introduction

The calorimeters, thermopiles, and bolometers are electrical substitution radiometers and operate at ambient temperature. They provide uncertainties between 0.1% and 0.3% are not rapids, and they present thermal time constants of about few minutes [1]. Their performances are essentially limited by the materials' thermal properties at room temperature. Those detectors are thermal ones, in which the incident radiation for the spectral range of 200 to 2000 nm is converted into heat. The resulting diffuse heat causes a temperature change, which it can be measured using a thermal sensor.

Over the past few years, the most electrical substitution radiometers operate at 4.2 K and are known as cryogenic radiometers. Hence, they became national primary standards for radiant power. The operation at liquid helium temperature reduces non-equivalence errors between electrical and radiative heating by increasing the receiver's thermal conductivity and eliminating heater lead losses through the use of a superconducting wire [2,3]. The low specific heat of cooled copper enables construction of geometrical ‘closed’ black body cavity with high absorption and without thermal constant rise.

In this paper, we develop chemical, optical, and thermal studies in order to control all the parameters having an influence on the design of the laser absorbing cavity. In our case, the last study will be conducted at the bottom of a cryostat maintained at nitrogen liquid temperature.

### Blackening absorber method

The cavity of our radiometer is manufactured on pure copper, whose inside surface will be blackened to receive the light radiation. For thermal detectors, the absorbing cavity surface is blackened in order to increase its absorbance without considerably raising the cavity dimensions. In fact, the detector thermal time constant essentially depends on its real dimensions. Many techniques are used to blacken the surfaces which receive incident radiant power, like black painting, graphite-black coating, and black gold coating. However, all these techniques provide very low mechanical strength coating and we can observe damages after work cycles of heating-cooling.

As a consequence, we used an electrochemical method to blacken the inner surface of the absorbing cavity. This technique provides two copper oxides, the CuO and the Cu_2_O. Copper oxide is a semiconductor that has been studied for photothermal, photoconductive, and photochemical applications [4-6].

In our case, we have used electrochemical route to obtain these oxide coatings on cooper samples. As the anodic oxidation consists of immersing the material in a specific chemical solution with an applied current-voltage during a given time, we have developed a study of the parameters influencing the anodization process. This was done in order to select the adequate combination which gives a high absorbance of the laser cavity.

This study essentially concerns the chemical solution concentrations to be used, the current and voltage values to be applied, and the necessary attack time for oxidation.

**Figure 1:**
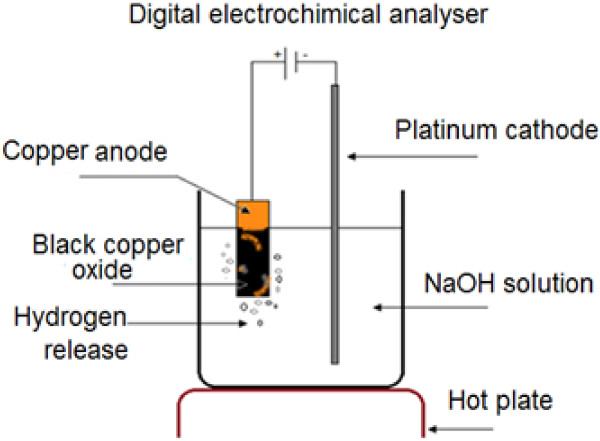
Experimental process of cupic oxide development.

In order to obtain a good uniformity of absorbing the coating, all our copper samples are mechanically polished before being anodized.Figure 1 gives the different details of the used bench to anodize our samples. It is essentially constituted by three electrodes: a platinum cathode, an anode that supports our samples, and a reference electrode. These electrodes are connected to a potentiostat which delivers voltage and current necessary to the anodization process. The whole bench is automatically controlled using the software Voltamaster 2.

The oxidation process is identified by the oxidation peaks recorded in Voltamaster 2. The first peak indicates that the oxidized copper formed is Cu_2_O; the second one indicates the transformation of Cu_2_O on CuO.

In fact, copper oxidation is carried out in two steps:

(1) $$\text{Step}\;\left(1\right):\;2Cu\;+\;H_{2}O\;\to {Cu}_{2}O\;+2H+\;+2e‒$$

(2) $$\text{Step}\;\left(2\right):\;{Cu}_{2}O\;+\;H_{2}O\;\to \;2CuO\;+2H+\;+2e‒$$

**Figure 2:**
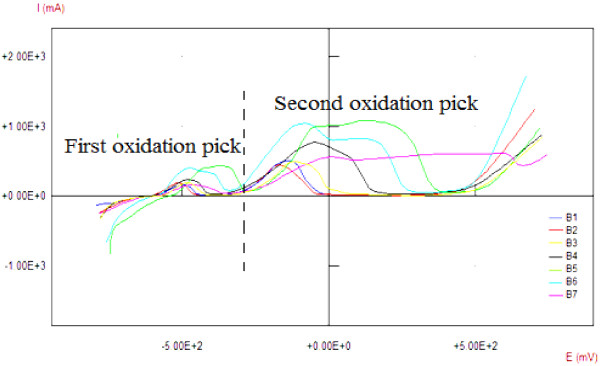
**Cyclic voltammograms obtained on copper samples of different surfaces *S*.** B1 {*S* =2 cm^2^}, B2 {*S* =4 cm^2^}, B3 {*S* =8 cm^2^}, B4 {*S* =16 cm^2^}, B5 {*S* =22 cm^2^}, B6 {*S* =36 cm^2^}, B7 {*S* =88 cm^2^}, in 2 mol L^-1^ NaOH solution with a slew rate =20 mV s^-1^.

We first studied the influence of the sample size on the oxidation peaks. In Table 1, we give the different dimensions of the samples studied. In Figure 2, we represent the oxidation process behavior, depending on the current and potential for different studied surfaces.From Figure 2, we notice that oxidation curves have the same behavior of the current density recorded for a period of tension imposed on the samples. We also remark that the interval of the oxidation potential is increasingly important for large areas.Besides this result, we can prove through Figure 2 that the oxidation is a surface phenomenon (specially oxidation curves of {C1, B2} and {C2, B3} samples). We can see that the amplitude of the oxidation current is insensitive to the variation of the sample thickness. Another important result found from this study is that the geometric shape of the sample affects the range of current and voltages oxidation.

**Table 1:** Geometry of the oxidized copper samples

**Sample reference**	**Surface (cm**^**2**^**)**	**Shape of the sample**	**Sample thickness (mm)**
B1	2	Rectangle	1
B2	4	Rectangle	1
B3	8	Rectangle	1
B4	16	Rectangle	1
B5	22	Rectangle	1
B6	36	Rectangle	1
B7	88	Hollow cylinder	1
C1	4	Rectangle	0.4
C2	8	Rectangle	0.4

The literature reviews showed that the value of the current density and the concentration of the solution anodization bath temperature affect more or less the morphological characteristics of the anodized films such as film thickness, pore diameter, intercellular distance, pore organization, or pore density [7]. This is why in our work, we study the influence of these factors on our samples. The various combinations used in our experimental process are summarized in (Table 2).

**Table 2:** Terms of oxidation of copper samples

**Sample reference**	**Oxidation method**	**Time (s)**	**Current (A)**	**Voltage (V)**
A	2	830	-	[-0.8; 0.8]
B	1	120	0.163	[-0.592; -0.385]
C	1	240	0.163	[-0.592; -0.385]
E	1	830	0.109	[-0.592; -0.385]
I	1	240	0.511	[-0.4; -0.064]
J	1	830	0.255	[-0.4; -0.064]
K	2	400	-	[-0.8; 0.8]
L	1	830	0.05	[-0.592; -0.385]
N	1	830	0.04	[-0.592; -0.385]
Q	1	360	0.163	[-0.592; -0.385]
R	2	240	-	[-0.8; 0.8]
R2	1	830	0.163	[-0.592; -0.385]

1, chronopotentiometric is an anodization technique using a fixed current density in a voltage interval of oxidation; 2, cyclic method using a variable current in a cycle of specified values.

### Optical characterization: reflectivity measurement

The spectral response of the obtained copper oxide samples using an electrochemical method was determined by ultraviolet-visible-infrared spectrophotometry. We define absorbance A(λ) factors as [8].

(3) $$A\left(\lambda \right)\;=\;1\;-R_{\text{total}}\left(\lambda \right)\;-\;T\left(\lambda \right)$$

where *R*_total_(λ) is the total reflection coefficient (specular and diffuse components); *T*(λ) is the transmission coefficient and in our case is considered equal to zero.

**Figure 3:**
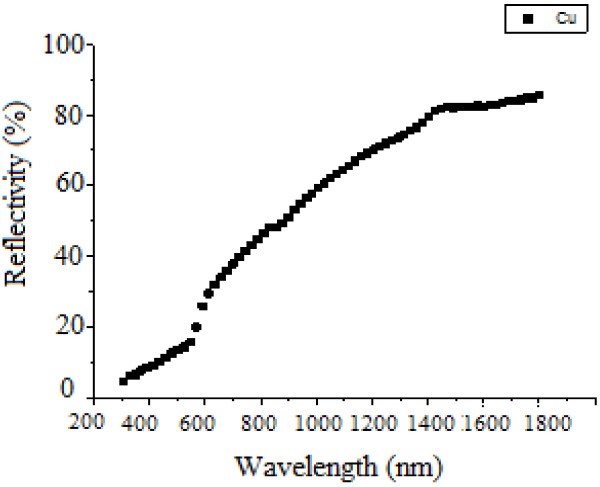
Reflectivity of non-anodized copper.

**Figure 4:**
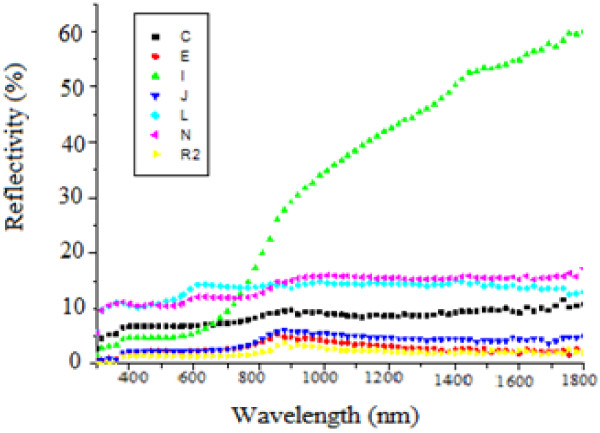
**Influence of the oxidation current value *I *on the reflectivity of anodized samples.** J {*I* =0.255 A; *t* =830 s}, E {*I* =0.109 A; *t* =830 s}, L {*I* =0.05 A; *t* =830 s}, N {*I* =0.04 A; *t* =830 s}, and R2 {*I* =0.163 A; *t* =830 s}, I {*I* =0.511 A; *t* =240 s}, C {*I* =0.163 A; *t* =240 s}.

**Figure 5:**
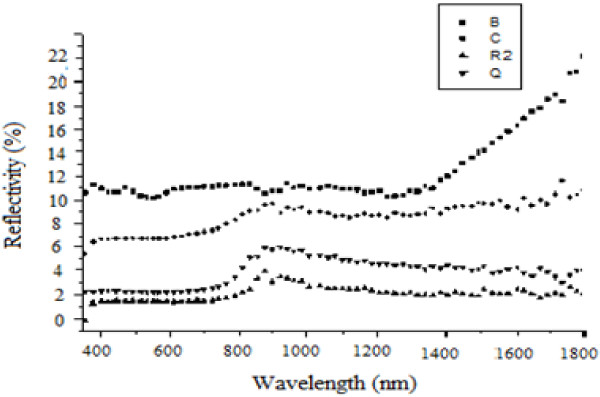
**Influence of chemical attack time *t *on the reflectivity of anodized sample by chronopotentiometric method.** B {*t* =120 s}, C {*t* =240 s}, R2 {*t* =830 s} and Q {*t* =360 s}.

**Figure 6:**
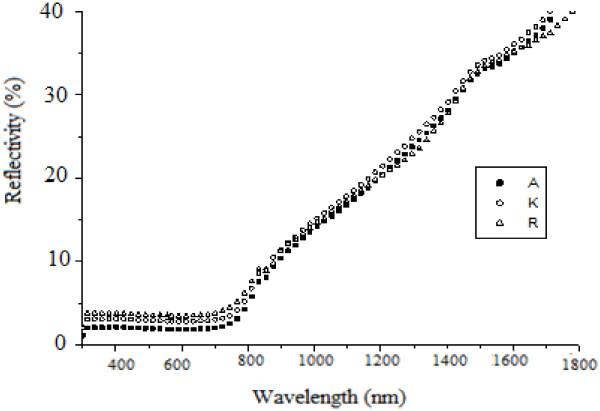
**Influence of the chemical attack time *t *used in the oxidation process of copper samples.** Influence of the chemical attack time *t* used in the oxidation process of copper samples anodized by cyclic method on its surfaces reflectivity; A {*t* =830 s}, K {*t* =400 s}, R {*t* =240 s}.

Figure 3 shows reflectivity of the non-anodized copper. Figures 4, 5, and 6 show that copper oxide has reduced reflectivity especially in IR range compared to the non-anodized copper one (Figure 3). We also note that the samples oxidized by a low-amplitude current of the first peak (Figure 2) have the same optical behavior characterized by an almost constant reflectivity over the hole-studied optical range {Figure 5; curves E, L, N, and R2}, the absorption edges of these samples was the same (about 850 nm). A similar result is recorded for a low current selected in the curve of the second oxidation peak {Figure 6, curve (E)}; this behavior is characteristic of the oxide Cu_2_O.

For the samples oxidized by a constant current method, we note that the absorbance is better when working in conditions of the first oxidation peak (shown in Figure 2) and when the value of the oxidation current is maximal (*I* =0.163 A). However, when we oxidize copper with the max current of the second peak {Figure 4, curve I} we notice that from 700 nm, the reflectivity increases with the wavelength from 6% to 60%, and the same behavior is also observed for samples oxidized by cyclic method (Figure 6, curves A, K, and R), it is the optical behavior characteristic of the oxide CuO [5]. In fact, the use of high current for oxidation, whatsoever when we oxidized with constant current or when we reached these values with the cyclic method, produces copper(II) oxide, so the obtained substrates have a reflectivity witch increases considerably after the absorption edge.

In conclusion, the current value of the oxidation process gives the composition of the oxide made on oxidized samples, in other term, it fixes the behavior of the optical characteristic of the obtained substrates.We noted also that the increase of the chemical attack time increases the absorption of substrates {Figure 5 samples (B, C, Q, and R2)}; in fact, the increase of the oxidation time enhances the number of pores and amplify its depths and as a consequence, the quantity of absorbed beam increases.

As the produced oxide will represent the absorbing layer of the thermal detector, it must have a constant reflectivity in the range of wavelengths to be calibrated and with the lowest possible value.

As result of this optical study, we can suppose that the experimental parameters to be chosen in our case are those of the following:

The oxidation current value is equal to the current value of the first oxidation peak, *I* =0.163 A.

The duration attack time of about 830 s; if we increased the time over 830 s, the formed oxide is dissolved in the solution (and we paid attention to the oxide reduction).

The temperature of the bath is about 90°C.

### Structural characterization of the coating

To understand the optical behavior of the copper oxide formed by the two methods and to confirm the advanced hypotheses in the preceding paragraph, we developed an X-ray study on our samples.

**Figure 7:**
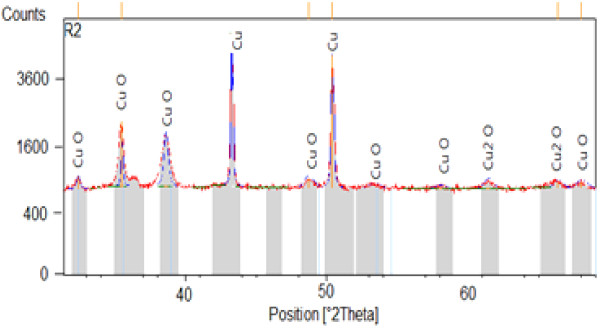
X-ray diffraction pattern of the sample (R2).

**Figure 8:**
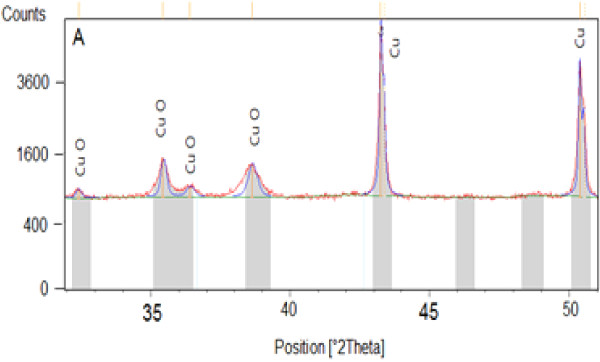
X-ray diffraction pattern of sample (A).

Diffraction patterns on some samples oxidized by different methods show the existence of three compounds: copper ‘Cu’, the copper oxide I (cuprite) ‘Cu_2_O’, and the copper(II) oxide (tenorite) ‘CuO’. The analysis shows that the copper used is a pure copper, and the samples oxidized by cyclic method (A, K, and R) and the samples oxidized with chronopotentiometric method high-current density are composed of copper and tenorite. The samples oxidized by chronopotentiometric method low-current density (B, C, E, J, L, N, and R2) are trained on copper, tenorite, and cuprite. The examples of X-ray characterization are shown in Figures 7 and 8.

Through these results, we can explain the reflectivity constancy of the samples prepared by chronopotentiometric method is mainly due to the presence of the copper(I) oxide.

### Copper oxide thermal diffusivity

In our case, the optical radiation is absorbed on the obtained copper oxide black coating and causes a temperature gradient across a heat link to a heat sink. The electrical power is then adjusted to give a similar temperature rise. Unfortunately, the equivalence between electrical heating and radiative heating cannot be obtained on the obvious way. In fact, the radiative heating is applied to the blackened front surface while the electrical one is generated on the outer absorbing surface cavity. As a result, we do not have the same heat wave propagation by the two heating procedures. To minimize this heat gradient, we have to use high thermal conductivity and diffusivity materials.

**Figure 9:**
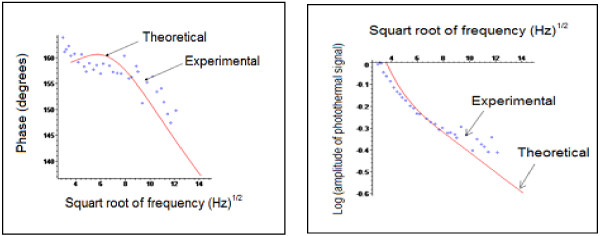
Theoretical and experimental photothermal signal for sample R2.

In order to determine the thermal properties of the obtained oxidized samples, we have used the ‘mirage effect’ photothermal technique [9-12]. Figure 9 shows the theoretical and experimental photothermal phase and amplitude variation as a function of frequency square root.

The obtained thermal diffusivity and conductivity of our optimum (sample R2) are respectively (11.5 ± 0.5) × 10^-7^ m^2^ s^-1^ and (370 ± 20) Wm^-1^ K^-1^, so we can consider that our substrate is a good thermal conductor.

### Design

The previous studies show that the best experimental parameters' combination to be considered is a 0.163 A current value and 830 s as attack time. Those conditions give samples with a reflectivity of about 3%. In order to reduce this reflectivity, we decided to consider a trap geometrical cavity in which the incident radiation must undergo many reflections before escaping from the detector (Figure 7). In this sense, we choose a design of the absorbing cavity like a cylinder machined at an angle of α.

**Figure 10:**
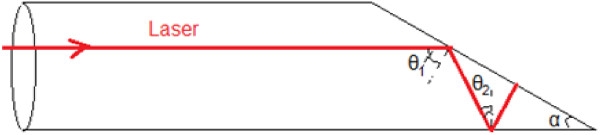
Simplified cross section of the oxidized copper trap detector.

Figure 10 illustrates the principle of the radiation propagation in the trap detector. We note ‘θi’ the angle formed by the laser beam and the normal to the surface at the *i*th reflection.

For our considered geometrical cavity and when we suppose that interior surface gives specular reflections, the reflection number is given by the following equation:

(4) $$n=\;\left(int\;\left\{\frac{\pi }{2a}\right\}\;-1\right)$$

where int is the integer part.

**Figure 11:**
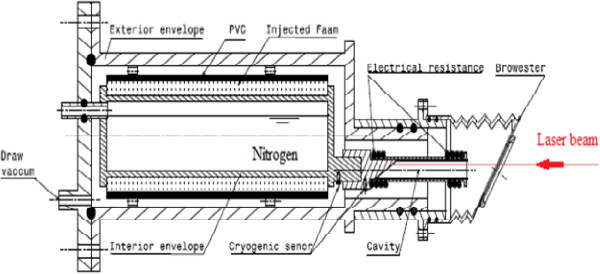
Design of the cryogenic radiometer.

When we choose an angle α of 30°, we obtain five reflections of the incident beam before escaping the cavity. Knowing that the inner surface have a reflectivity coefficient *ρ* at about 3%, the total coefficient of absorbed radiation before exit (1 - *ρ*^*n*^) is equal to 0.999999. Compared with the international references for absolute laser power measurements, our cavity can be considered as a good absorbing cavity [3,13-15].Our cryogenic radiometer is an electrical substitution detector operating at liquid nitrogen temperature (77 K). A schematic side viewing of our cryogenic radiometer is shown in Figure 11. The radiometer is designed specifically to accept collimated laser beam. The window port is tilted at Brewster's angle to minimize reflective losses when the laser beam is linearly polarized. The radiation-receiving cavity is a horizontal cylinder which is constructed from a copper tube containing an inclined plane. This cavity is thermally linked to the nitrogen liquid Dewar, and a black copper oxide makes its inner surface.

## Conclusions

The primary goal of our study is to find out the best absorbing cavity to be used as electrical substitution detector working at liquid nitrogen temperature. For this purpose, we developed different studies to optimize chemical, optical, and thermal parameters influencing the realized coatings. The obtained results are very encouraging. In one side, the selected shape of the cavity gives in theory a value of 0.9999999 as device absorbance. In another side, for 0.163 A current value and 830 s as attack time, the obtained coating has a reflectivity of about 3% in the spectral range from 300 to around 1,800 nm. This coating has an average roughness of about 85 nm and a thermal conductivity and diffusivity equal respectively to (370  ±  20) Wm^- 1^ K^- 1^ and (11.5 ±0.5) ×10^-7^ m^2^ s^-1^. These last values can be improved, when we operate at liquid nitrogen temperature below than 100 K.

The chosen cavity form gives 0.9999999 as device absorbance.

These encouraging characteristics allow us to grow the cavity dimensions without enhancing the thermal time constant.

In the future, we will definitely realize our cavity and we will develop an optical and a metrological study in order to determine experimentally the cavity absorbance and the absolute and spatial responsivities and the linearity range.

## Competing interests

The authors declare that they have no competing interests.

## Authors’ contributions

SB is involved in the design and characterization of the absorbing cavity and undertook the acquisition and the analysis of data. ZB is responsible for the analysis and interpretation of optical data, and structural study results, also revision of the manuscript. KT contributed in the conception and design of the cooling system (the cryogenic cavity). OT supervised the overall scheme of the radiometer and revision of the manuscript. All authors read and approved the final manuscript.

## Authors’ information

SBS is a PHD student in the Research Laboratory ‘Materials, Measurement and Applications,’ INSAT, Tunisia. ZB is an assistant professor in INSAT, Tunisia. KT is a Master's degree student in the Materials, Measurement and Applications, INSAT, Tunisia. OT is a professor in INSAT, Tunisia.
